# Escaping introns in COI through cDNA barcoding of mushrooms: *Pleurotus* as a test case

**DOI:** 10.1002/ece3.3049

**Published:** 2017-07-29

**Authors:** Farhat A. Avin, Bhassu Subha, Yee‐Shin Tan, Thomas W. A. Braukmann, Sabaratnam Vikineswary, Paul D. N. Hebert

**Affiliations:** ^1^ Mushroom Research Centre (MRC) University of Malaya Kuala Lumpur Malaysia; ^2^ Department of Biotechnology Faculty of Science Lincoln University College 47301 Petaling Jaya Malaysia; ^3^ Centre for Biotechnology in Agriculture Research (CEBAR) Division of Genetics and Molecular Biology University of Malaya Kuala Lumpur Malaysia; ^4^ Institute of Biological Sciences Faculty of Science University of Malaya Kuala Lumpur Malaysia; ^5^ Centre for Biodiversity Genomics University of Guelph Guelph ON Canada

**Keywords:** COI, DNA barcoding, internal transcribed spacer, oyster mushrooms, taxonomic verification

## Abstract

DNA barcoding involves the use of one or more short, standardized DNA fragments for the rapid identification of species. A 648‐bp segment near the 5′ terminus of the mitochondrial cytochrome *c* oxidase subunit I (COI) gene has been adopted as the universal DNA barcode for members of the animal kingdom, but its utility in mushrooms is complicated by the frequent occurrence of large introns. As a consequence, ITS has been adopted as the standard DNA barcode marker for mushrooms despite several shortcomings. This study employed newly designed primers coupled with cDNA analysis to examine COI sequence diversity in six species of *Pleurotus* and compared these results with those for ITS. The ability of the COI gene to discriminate six species of *Pleurotus*, the commonly cultivated oyster mushroom, was examined by analysis of cDNA. The amplification success, sequence variation within and among species, and the ability to design effective primers was tested. We compared ITS sequences to their COI cDNA counterparts for all isolates. ITS discriminated between all six species, but some sequence results were uninterpretable, because of length variation among ITS copies. By comparison, a complete COI sequences were recovered from all but three individuals of *Pleurotus giganteus* where only the 5′ region was obtained. The COI sequences permitted the resolution of all species when partial data was excluded for *P. giganteus*. Our results suggest that COI can be a useful barcode marker for mushrooms when cDNA analysis is adopted, permitting identifications in cases where ITS cannot be recovered or where it offers higher resolution when fresh tissue is. The suitability of this approach remains to be confirmed for other mushrooms.

## 1 INTRODUCTION

DNA barcoding employs short, standardized DNA fragments for the rapid identification of species (Gilmore, Graefenhan, Louis Seize, & Seifert, 2009; Hebert, Cywinska, & Ball, 2003; Nguyen & Seifert, 2008; Vialle et al., 2009). This approach is particularly valuable for verifying species identification, and for the evaluation of taxonomic diversity in organisms with cryptic morphology such as fungi (Dentinger, Didukh, & Moncalvo, 2011). The use of molecular tools is essential for identifying and classifying the 90%–95% of undescribed fungi (Blackwell, 2011; Seifert, 2009); The ribosomal internal transcribed spacer (ITS), a highly variable region between the conserved sequences of the small subunit, 5.8S, and large subunit rRNA genes, has been adopted as the primary DNA barcode marker for fungi (Schoch, Seifert, Huhndorf, et al., 2012).

The ideal DNA barcode region is easy to amplify and variable enough to discriminate species, a condition that is best met when variation within species is low and divergence between species is high, a situation which creates a “barcode gap” (Hebert et al., 2003; Lahaye et al., 2008). A 648‐bp segment near the 5′ terminus of the mitochondrial cytochrome *c* oxidase subunit I (COI) gene has been adopted as the DNA barcode region for animals because its performance in species discrimination is high and it is usually easy to recover (Hebert et al., 2003). Contrary to animals, no single gene region has been found that serves as an ideal DNA barcode for fungi and plants. As a consequence, a multi‐locus barcode approach has been adopted to improve resolution across plants and fungi (Hollingsworth et al., 2009; James et al., 2006), and ITS has been adopted as the standard barcode region for fungi (Avin, Bhassu, Shin, & Sabaratnam, 2012; Begerow, Nilsson, Unterseher, & Maier, 2010; Schoch, Seifert, Huhndorf, et al., 2012; Seifert, 2009) although studies have shown that this gene region often fails to distinguish closely related fungal species (Schoch, Seifert, Caldeira, et al., 2012). Despite the acceptance of ITS as the fungal barcode, length variation in this region makes sequence alignment difficult across divergent taxa (Dentinger et al. (2011). Additional markers beyond ITS are needed for fungal barcoding, but finding suitable loci that can be easily amplified across the diversity of fungi remains a challenge (Robert et al., 2011; Stielow et al., 2015). COI has potential to address this gap because alignment of this locus across a divergent set of taxa is trivial (Dentinger et al., 2011).

A few studies have compared the resolution of ITS and COI in sets of closely allied species. COI was more effective than ITS in *Penicillium* (Seifert et al., 2007), while COI and ITS were equally effective in *Leohumicola*, (Nguyen & Seifert, 2008). In the Agaricomycotina, COI and ITS generally delivered similar resolution, but the prevalence of introns resulted in COI not being recovered from many taxa (Dentinger et al., 2011). Conversely, COI sequences showed low divergences in *Fusarium* (Gilmore et al., 2009) and *Aspergillus* (Geiser et al., 2007), although data interpretation was complicated by the apparent presence of multiple copies of COI, perhaps reflecting the recovery of nuclear pseudogenes. The strong performance of COI as a DNA barcode in animals (Hebert et al., 2003) suggests the value of exploring its use as a marker in mushrooms. Similar to the multi‐locus barcode approach used in plants, COI could be used in conjunction with ITS for the identification of fungal species. There is one barrier to the implementation; the prevalence of introns in the COI gene of many fungal species including mushrooms is well documented (Seifert, 2009; Vialle et al., 2009). For example, nine introns occur in *Pleurotus ostreatus* (Wang, Zeng, Hon, Zhang, & Leung, 2008), 19 in *Agaricus bisporus* (Férandon et al., 2010), 15 in *Trametes cingulata* (Haridas & Gantt, 2010) and four in *Agrocybe aegerita* (Gonzalez, Barroso, & Labarère, 1998). These introns are often long, leading to extreme variation in length of the COI gene from approximately 1,584 bp in species lacking introns to over 22 kb in those with many introns (Férandon et al., 2010; Gonzalez et al., 1998; Haridas & Gantt, 2010; Wang et al., 2008). The presence of these introns impedes sequence recovery by conventional PCR (Seifert, 2009; Seifert et al., 2007), a factor which has supported the adoption of ITS as the sole DNA barcode for mushrooms (Schoch & Seifert, 2012; Vialle et al., 2009).

Although COI seems to have the potential to reliably identify taxa, there is a need for more detailed study. In particular, given the prevalence of introns and the apparent occurrence of nuclear pseudogenes, it is critical to adopt RT‐PCR to properly recover and evaluate the capacity of COI sequences to resolve fungal species.

The genus *Pleurotus* (Jacq. Fr.) Kummer (Basidiomycotina, Pleurotaceae) is a cosmopolitan group of edible mushrooms prized for their antibiotic, antiviral, anticholestorilic, and anti‐tumor properties (Pawlik, Janusz, Koszerny, Małek, & Rogalski, 2012). Because of its high diversity, *Pleurotus* has taxonomic problems that reflect the absence of type specimens, the instability of morphological characters, and insufficient information on physiological characteristics and mating compatibility (both wild and cultivated strains) (Avin, Bhassu, Tan, Shahbazi, & Vikineswary, 2014; Pawlik et al., 2012; Vilgalys, Moncalvo, Liou, & Volovsek, 1996; Zervakis & Balis, 1996). These taxonomic and identification issues within *Pleurotus* have prompted investigations using molecular markers to identify strains and species (Avin, Bhassu, Rameeh, Tan, & Vikineswary, 2016; Bao, Ishihara, Mori, & Kitamoto, 2004).

In this study, we examine the ability of the COI gene to discriminate six species of *Pleurotus*. We test amplification success, sequence variation within and among species, and the ability to design effective primers. We also recover ITS sequences from all isolates to allow their comparison with the sequences recovered through the analysis of cDNA from COI.

## 2 MATERIALS AND METHODS

### 2.1 Sample collection

The 24 strains examined in this study included representatives of six species of *Pleurotus* (Table 1). They were mostly obtained from mushroom farms in Malaysia or from the University of Malaya collection. A few isolates were newly collected from Malaysia, while others were imported from China or Iraq (Table 1). The species assignment for each isolate was verified by comparison of morphological traits of basidiocarps and mycelial cultures.

**Table 1:** List of species and strains used in this study and length of amplicons for COI and ITS. Bold process IDs for the samples sequenced in this are also indicated and are publically available

	Strain ID	Species	Source	Length of amplicon (bp)				Sequence ID (The BOLD System)	NCBI GenBank accession number		
				COI			ITS		ITS	FL(COI	3′)COI
				5′	3′	Full length					
1	FUM‐077	*Pleurotus pulmonarius*	Farm Malaysia	759	757	1,516	592	CDB0001‐15	KY951484	KY951528	KY951506
2	FUM‐078	*Pleurotus pulmonarius*	Farm Malaysia	759	757	1,516	592	CDB0002‐15	KY951490	KY951534	KY951512
3	FUM‐079	*Pleurotus pulmonarius*	Farm Malaysia	759	757	1,516	592	CDB0003‐15	KY951489	KY951533	KY951511
4	FUM‐080	*Pleurotus giganteus*	Wild Malaysia	759	Partial	X	613	CDB0004‐15	KY951479	X	X
5	FUM‐081	*Pleurotus ostreatus*	Iraq	759	757	1,516	602	CDB0005‐15	KY951483	KY951527	KY951505
6	FUM‐082	*Pleurotus ostreatus*	Farm Malaysia	759	757	1,516	X	CDB0006‐15	X	KY951520	KY951498
7	FUM‐084	*Pleurotus giganteus*	Wild Malaysia	759	Partial	X	618	CDB0007‐15	KY951477	X	X
8	FUM‐085	*Pleurotus flabellatus*	Iraq	759	757	1,516	625	CDB0008‐15	KY951474	KY951516	KY951494
9	FUM‐086	*Pleurotus ostreatus*	UM collection	759	757	1,516	606	CDB0009‐15	KY951482	KY951525	KY951503
10	FUM‐087	*Pleurotus ostreatus*	Iraq	759	757	1,516	X	CDB0010‐15	X	KY951523	KY951501
11	FUM‐088	*Pleurotus citrinopileatus*	Farm Malaysia	759	757	1,516	597	CDB0011‐15	KY951471	KY951513	KY951491
12	FUM‐089	*Pleurotus pulmonarius*	Farm Malaysia	759	757	1,516	592	CDB0012‐15	KY951488	KY951532	KY951510
13	FUM‐090	*Pleurotus ostreatus*	China	759	757	1,516	602	CDB0013‐15	KY951480	KY951521	KY951499
14	FUM‐091	*Pleurotus pulmonarius*	Farm Malaysia	759	757	1,516	592	CDB0014‐15	KY951487	KY951531	KY951509
15	FUM‐093	*Pleurotus flabellatus*	Farm Malaysia	759	757	1,516	625	CDB0015‐15	KY951475	KY951517	KY951495
16	FUM‐095	*Pleurotus citrinopileatus*	Farm Malaysia	759	757	1,516	596	CDB0016‐15	KY951473	KY951515	KY951493
17	FUM‐096	*Pleurotus pulmonarius*	Farm Malaysia	759	757	1,516	592	CDB0017‐15	KY951486	KY951530	KY951508
18	FUM‐099	*Pleurotus giganteus*	China	759	Partial	X	610	CDB0018‐15	KY951478	KY951519	KY951497
19	FUM‐100	*Pleurotus ostreatus*	Farm Malaysia	759	757	1,516	609	CDB0019‐15	KY951481	KY951524	KY951502
20	FUM‐101	*Pleurotus ostreatus*	Farm Malaysia	759	757	1,516	X	CDB0020‐15	X	KY951526	KY951504
21	FUM‐102	*Pleurotus ostreatus*	UM collection	759	757	1,516	X	CDB0021‐15	X	KY951522	KY951500
22	FUM‐103	*Pleurotus pulmonarius*	UM collection	759	757	1,516	592	CDB0022‐15	KY951485	KY951529	KY951507
23	FUM‐104	*Pleurotus eryngii*	UM collection	759	757	1,516	600	CDB0023‐15	KY951476	KY951518	KY951496
24	FUM‐105	*Pleurotus citrinopileatus*	UM collection	759	757	1,516	598	CDB0024‐15	KY951472	KY951514	KY951492

### 2.2 DNA and RNA extraction and cDNA synthesis

Total genomic DNA was extracted from fresh mycelium by a rapid protocol (Avin, Bhassu, & Sabaratnam, 2013). Briefly, after adding sufficient 2% SDS buffer, the samples were homogenized at 65°C for 30 min. The mixture was purified twice with phenol: CHCl_3_: Isoamyl alcohol (25: 24: 1). DNA was precipitated with cold isopropanol, and then pelleted by centrifugation at 4°C for 15 min at 11,000 × g. The resultant DNA pellet was dissolved in TE buffer and stored at ‐20°C.

Total RNA was isolated from fresh mycelium using Trizol (Invitrogen, USA). Briefly, sufficient Trizol was added to the homogenized mycelia and incubated at 25°C for 15 min, then purified by chloroform. RNA was precipitated by cold ethanol and the pellet was washed twice with 70% ethanol. The RNA pellet was then dissolved in RNAase free water and stored at ‐80°C. Samples that did not successfully amplify in the first round of RT‐PCR were re‐extracted using Nucleospin^®^ RNA columns (Macherey‐Nagel, Germany) following the manufacturers protocol. This included a DNAase treatment prior to elution in nuclease free water.

Total cDNA was synthesized from the RNA extracts using an Access One Step RT‐PCR system kit (Promega, USA). The first mixture was generated by gently mixing 1.0 μl of total extracted RNA, 1.0 μl of Oligo dt primer, and 3.0 μl of Nuclease‐free H_2_O that was incubated for 5 min at 70°C. The second mixture was prepared by mixing 6.1 μl of Nuclease‐free H_2_O, 4.0 μl of Improm II reaction buffer, 2.4 μl of 25 mmol/L MgCl_2_, 1.0 μl of 10 mmol/L dNTP mix, 0.5 μl of RNAsin ribonuclease inhibitor and 1.0 μl of Improm II reverse transcriptase. Mixtures I and II were then combined for each sample and incubated for: 5 min at 25°C, 60 min at 42°C, and 15 min at 70°C before being stored at ‐20°C.

### 2.3 Primer design

**Figure 1:**
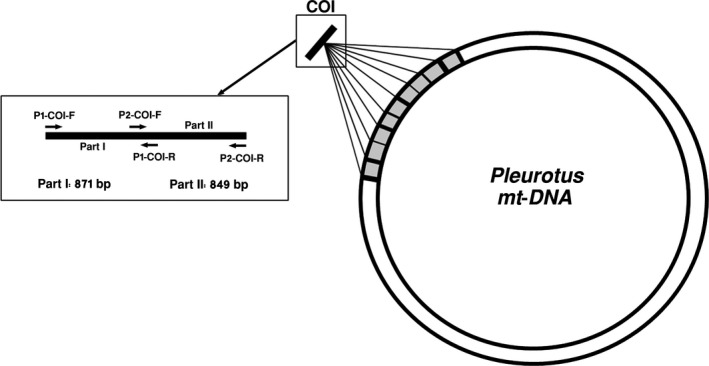
Schematic map of *Pleurotus ostreatus* mitochondrial COI gene. Grey areas indicate the positions of the nine introns while the black bands designate exons. The left box shows the location and orientation of the primers on the open reading frames of COI

The coding sequence of COI from the mitochondrial genome of *P. ostreatus* (19: EF204913) was used as a reference to design primers (Figure 1). Several criteria, including the generation of proper length fragments (800–900 bp) with enough conserved sites in the binding regions were employed to design primers. NCBI Primer‐BLAST was used to design primer pairs for two cDNA regions that spanned the coding sequence of COI (Rozen & Skaletsky, 2000; Ye et al., 2012). Figure 1 shows the location and orientation of these primers on the open reading frames of COI. Primer ID, sequence and annealing temperatures are provided in Table 2.

**Table 2:**
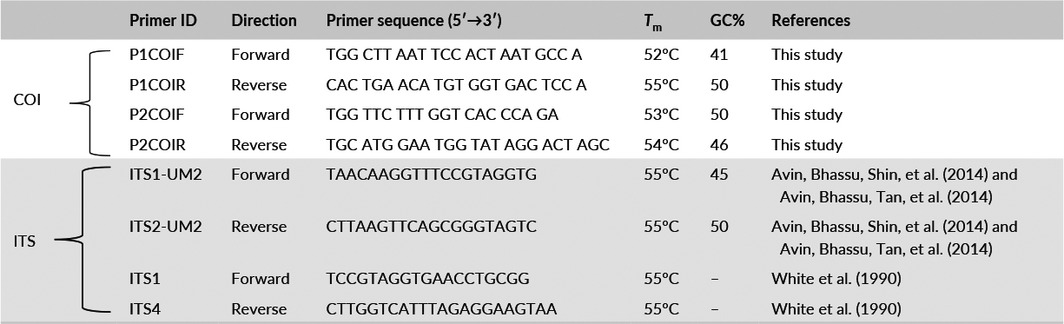
List of the primers used for amplification of COI and ITS from *Pleurotus* species

### 2.4 PCR and reverse transcription (RT)‐PCR conditions

PCR amplification of the COI cDNA employed an initial denaturation at 95°C for 5 min; followed by 30 cycles with denaturation at 94°C, annealing at 55°C and extension at 72°C for 1 min; followed by a final extension at 72°C for 10 min. The 50 μl PCR reactions included 4.0 μl (~100 ng) of template DNA (cDNA), 1.0 mmol/L MgCl_2_, 0.4 μmol/L of each primer (Part I: P1COIF & P1COIR and Part II: P2COIF & P2COIR), 0.4 mmol/L of each dNTP, 10 μl of 5× *Taq* buffer, 2 units of Go*Taq* ^®^ Flexi DNA Polymerase (Promega, USA). We used genomic DNA to amplify and sequence the ITS region with primers ITS1 and ITS4 using standard protocols (White, Bruns, Lee, & Taylor, 1990), or with local primers ITS1‐UM2 and ITS2‐UM2 (Avin, Bhassu, Shin, & Vikineswary, 2014). Successfully amplified PCR products were purified using the Nucleospin Extract II Kit (Chemopharm), and bidirectionally sequenced using an ABI 3730XL automated sequencer. Sequences along with voucher information were deposited in the Barcode of Life Data System (BOLD Process IDs; CDB001‐CDB024‐15) (Ratnasingham & Hebert, 2007) and are publicly available in NCBI GenBank (Table 1).

### 2.5 Sequence alignment, barcode gap analysis, and phylogenetic analysis

Chromatograms were edited using ChromasPro version 1.7.6 (Technelysium Pty Ltd., QLD, Australia). Additional ITS sequences were retrieved from GenBank and included in our analysis (Accession Numbers AF465404, EU314927, HM590443, EF514248, KF681359, EU424300, KJ862075, KC582641, KJ862073, JQ026939, EU233951, AY696300, AY450349, HM245782, KP120919, KP012913, KF724509, JN043316, KC582636, JQ837487, EU424288 and AY265827). The sequences were aligned using MEGA ver. 6.0 (Pawlik et al., 2012) and BioEdit ver. 7.2.5 (Hall, 1999). Barcode gap analysis was performed in BOLD 3 (Ratnasingham & Hebert, 2007) under a Kimura two‐parameter model with pairwise deletion of gaps and ambiguities on complete COI and ITS sequences. MEGA 6 (Tamura, Stecher, Peterson, Filipski, & Kumar, 2013) was used to determine the model that best fit sequence evolution for COI and ITS datasets prior to phylogenetic tree construction using maximum likelihood (ML). Phylogenetic trees were constructed for COI data both with and without partial sequences (5′ end) for *Pleurotus giganteus*. ITS trees were constructed with only sequences obtained in this study, and after including GenBank sequences for the species in our data sets. According to Bayesian information criterion (Schwarz, 1978), a General Time Reversible model (Lanave, Preparata, Sacone, & Serio, 1984) with rate variation among nucleotides following a discrete gamma distribution (GTR+G) was selected as the best fit model for COI data, and a Tamura three parameter model (Tamura, 1992) with a proportion of invariable sites (TN92+I) for ITS data (with and without GenBank sequences). For both genes, ML trees were constructed in MEGA 6 (Tamura et al., 2013) under the selected model; branch topology was optimized using extensive subtree pruning and regrafting (SPR) with branch swap filter selected. The stability of nodes was inferred by non‐parametric bootstrapping (Felsenstein, 1985), using 1,000 heuristic bootstrap pseudoreplicates. DNAsp ver. 5.10 was used to calculate the haplotype data file and genetic divergences (Librado & Rozas, 2009). To estimate the significance of variance within and among species, an AMOVA (analysis of molecular variance) was calculated using Arlequin ver. 3.50 (Excoffier, Laval, & Schneider, 2005).

## 3 RESULTS

**Figure 2:**
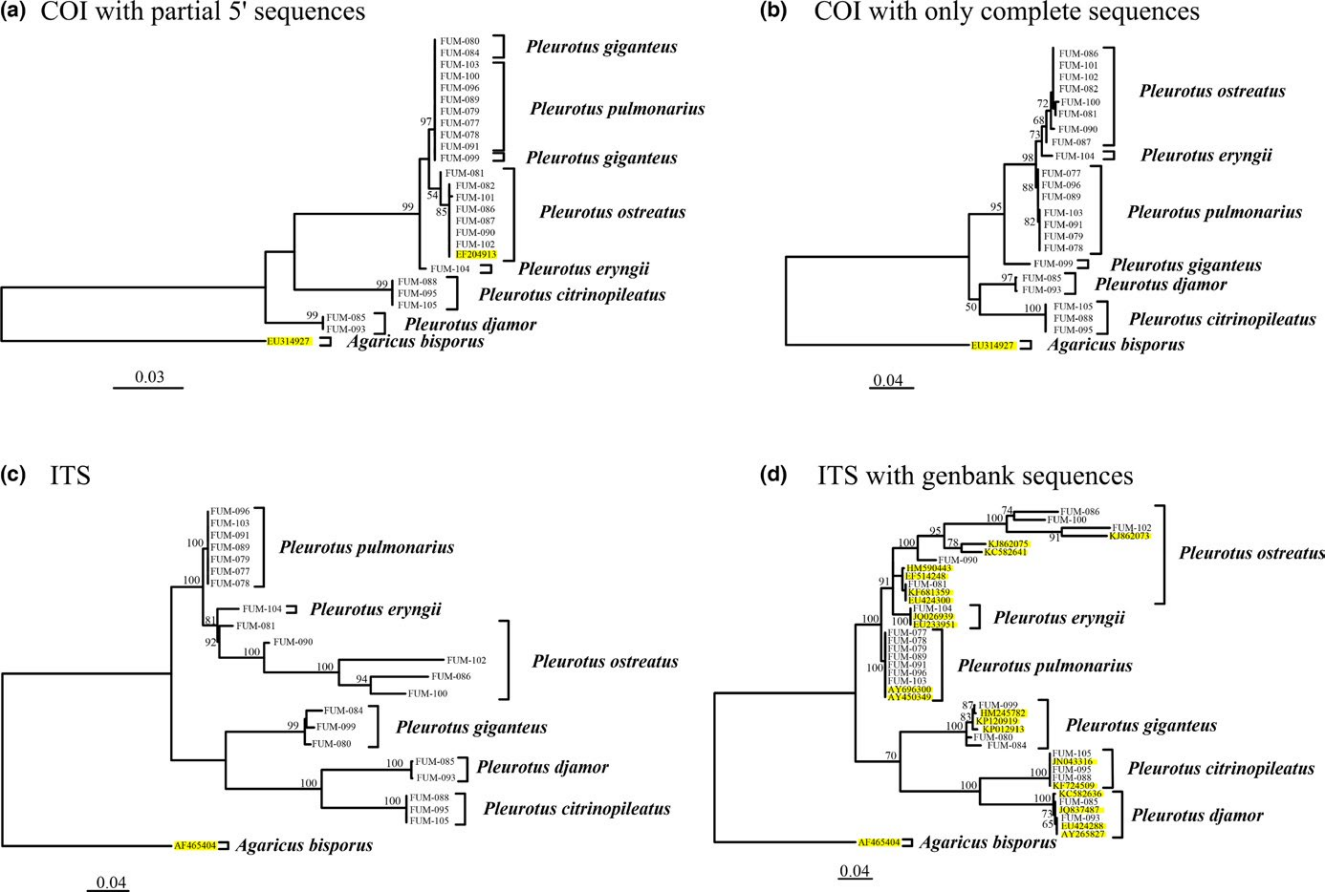
COI and ITS phylogenetic analyzes. (a–d) Phylogeny reconstruction based on maximum likelihood under a GTR+G model for COI and a TN92+I model for ITS. Numbers at the nodes indicate the percentage of bootstrap replicates supporting a given topology, although bootstrap values below 50% are not indicated. Samples ID and species delimitations are indicated at the tips of the tree. One COI tree for *Pleurotus giganteus* is based on 759‐bp COI‐5′ fragments, while sequences for the other taxa were full length 1,516 bp. Twenty‐two ITS sequences and two mitochondrial sequences were retrieved from GenBank; they are indicated in yellow

An interpretable ITS sequence was recovered from 20 of the 24 specimens, including at least one representative of each species with sequences varying in length from 592 to 625 bp (Table 1). A COI sequence was recovered from all specimens, but only a partial COI‐3′ sequence was obtained from specimens of *P. giganteus*. Near full length COI sequences were generated by aligning and assembling a consensus of the 5′ and 3′ reads for the five species with reads for both regions (Table 1 and 3). Because the COI sequences were generated from cDNA template generated by RT‐PCR they lacked introns, while ITS was amplified using standard PCR (Figure 2 and Table 3).

**Table 3:** Comparison of four potential markers for DNA barcoding of *Pleurotus*

Region	COI‐5′	COI‐3′	COI‐whole	ITS
Method	RT‐PCR	RT‐PCR	RT‐PCR	PCR
No. of mushroom strains analyzed	24	24	24	24
No. with sequence record	24	21 + 3 partial	21	20
Number of introns	5	5	9	N/A
Number of exons	6	6	10	N/A
Final fragment length	759	757	1,516	592–625
Gap	No	No	No	Yes
Number of haplotypes	7	10	13	13
Conserved sites	583/759 (76.8%)	576/757 (76.1%)	1,165/1,516 (76.8%)	271/719 (37.7%)
Variable sites	176/759 (23.2%)	181/757 (23.9%)	352/1,516 (23.1%)	397/719 (55.2%)
Parsimony informative sites	87/759 (11.5%)	99/757 (13.1%)	186/1,516 (12.3%)	276/719 (38.4%)
Singleton sites	89/759 (11.7%)	82/757 (10.8%)	166/1,516 (10.9%)	122/719 (17.0%)
Total number of mutations	193/759 (25.4%)	205/757 (27.1%)	380/1,516 (25.1%)	390/719 (54.2%)
Overall mean distance	0.050	0.064	0.059	0.199
G+C content (%)	33.3%	34.4%	33.9%	43.4%

The percentage of variable sites for all six species was computed for both genes (Table 3). Across all 1,516 sites for COI, 76.8% were conserved, while 23.1% were variable with 12.3% being parsimony informative and 10.9% singletons. By comparison, 37.7% of the 715 ITS sites were conserved, while 55.2% were variable with 38.4% being parsimony informative, and 17.0% singletons (Table 3). Due to the indels in ITS, the mean divergence for all 20 sequences was higher for ITS (0.199) than for COI (0.059). Intra‐specific divergences were generally slightly higher for ITS than COI, but so too were inter‐specific divergences. Barcode gap analysis supports higher interspecific and intraspecific distances for ITS than COI. Both markers indicate *P. ostreatus*,* P. eryngii*, and *P. pulmonarius* are relatively close (Table 4) and fall under the 2% divergence threshold for COI and ITS (except *P. eryngii*). However, the use of the closely related mushrooms in our analysis with small sample sizes may explain the low divergence threshold (below 2%). The maximum intraspecific distance was greater for both COI and ITS in *P. ostreatus*. Otherwise, intraspecific distances were low for the remaining *Pleurotus* species with multiple representatives per species.

**Table 4:** Barcode gap analysis for COI and ITS. Species below the 2% threshold are indicated in bold

Species	Number of individuals	Mean intra‐sp	Max intra‐sp	Distance to NN	NN
COI					
*P. citrinopileatus*	3	0	0	7.7	*P. djamor*
*P. djamor*	2	0.13	0.13	7.7	*P. citrinopileatus*
*P. eryngii*	1	N/A	N/A	**1.47**	*P. ostreatus*
*P. giganteus*	1	N/A	N/A	4.55	*P. pulmonarius*
*P. ostreatus*	8	0.45	1.2	**1.2**	*P. pulmonarius*
*P. pulmonarius*	7	0.08	0.13	**1.2**	*P. ostreatus*
ITS					
*P. citrinopileatus*	3	0	0	12.06	*P. giganteus*
*P. djamor*	2	0.16	0.16	19.37	*P. pulmonarius*
*P. eryngii*	1	N/A	N/A	2.37	*P. ostreatus*
*P. giganteus*	3	2.33	2.68	12.06	*P. citrinopileatus*
*P. ostreatus*	4	12.18	17.89	**1.89**	*P. pulmonarius*
*P. pulmonarius*	7	0	0	**1.89**	*P. ostreatus*

Figure 2a–d shows ML trees for COI and ITS with bootstrap values for each node based on 1,000 replicates. ITS (Figure 2c,d) discriminated all six species with strong support, but sequences from four of eight specimens of *P. ostreatus* failed. COI sequences were recovered from all specimens, albeit just partial COI‐5′ sequences for *P. giganteus*. COI failed to distinguish between *P. pulmonarius* and *P. giganteus* when partial 5′ sequences were included, but when partial sequences were excluded, COI distinguished between these two species with strong support (Figure 2). Overall, both markers readily distinguished between species with moderate to strong support. When sampling was improved for ITS with Genbank sequences, there was strong support for the monophyly of the six *Pleurotus* species, results confirming our morphological identifications (Figure 2d).

## 4 DISCUSSION

In contrast to prior studies that failed to recover COI through conventional PCR‐based approaches (Dentinger et al., 2011; Vialle et al., 2009), the cDNA approach employed in this analysis recovered full COI sequences from all six species of oyster mushrooms (barring a few incomplete recoveries for *P. giganteus*). The past failures of standard PCR were undoubtedly due to the presence of several large introns in the COI gene of *Pleurotus* (Dentinger et al., 2011; Seifert, 2009; Seifert et al., 2007). However, cDNA barcoding escapes this problem, generating amplicons that are easily aligned. The present study generated a 1,516‐bp COI sequences from 21 of the 24 specimens, failing only to recover full sequence information from the 3′ region of *P. giganteus*. Our failure to amplify the 3′ end of *P. giganteus* reflects the need to further optimize COI primers for *Pleurotus* (and other mushrooms) given the diagnostic ability of the 3′ end of this gene. Alternatively, more samples of different species should be sequenced and aligned to design appropriate primer pairs on the most conserved regios. ITS sequences were recovered from all six species, but results from four of the 24 specimens were uninterpretable due to sequence length variation. Although the number of species examined in this study was small, the success of COI in discriminating these taxa justifies a larger‐scale effort to validate the effectiveness of COI as a barcode for *Pleurotus* and other mushrooms.

Paralogues (multiple copies) of COI and low success in species delimitation rate were reported in a study on the important pathogenic and commonly isolated *Fusarium* (Gilmore et al., 2009) and also in certain genera of the Agaricomycotina (Dentinger et al., 2011). These paralogues likely represent nuclear encoded pseudogenes of COI (Gilmore et al., 2009), and can be avoided using RT‐PCR because pseudogenes are not expressed. By contrast, the current study revealed evidence of sequence divergence among the multiple copies of ITS within single individuals that complicated sequence recovery from *Pleurotus ostreatus*. Sequence length variation was not encountered with COI sequences generated via cDNA. This is a key advantage that an RNA‐based approach has over conventional PCR for barcoding when fresh or cryopreserved tissue is available.

The primers employed in this study failed to amplify species in families (Marasmiaceae, Lyophyllaceae and Bolbitiaceae) closely related to the Pleurotaceae, indicating that the development of a universal primer set enabling COI recovery from mushrooms requires further work (Dentinger et al., 2011). However, studies on the animal kingdom have shown the feasibility of designing primer sets that are effective for most species within even the most species‐rich phylum, Arthropoda. More work on primer design, including the development of cocktails with degeneracy, could lead to the development of primer sets effective for broad assemblages of mushrooms.

Our work suggests that both ITS and COI are potentially effective DNA barcodes as they both share a similar capacity to delimit the species of *Pleurotus* examined in this study. The information that COI and ITS provided was complementary. For example, ITS sequences could not be recovered from four specimens of *P. ostreatus* that did generate COI data. Conversely, our primer set failed to recover full COI‐3′ sequence from three specimens of *P. giganteus* that generated ITS sequences. The loss of data at the 3′ end of COI hindered the ability of COI to distinguish between *P. giganteus* and *P. pulmonarius*. However, when partial sequences were excluded and the full COI sequenced was analyzed, COI distinguished all six species examined in this study. Our results support the conclusion that the 3′ end of COI is essential for resolving one pair *Pleurotus* species examined in this study. The ITS sequences from different species of *Pleurotus* contained indels, variation that often exists among conspecific individuals that can complicate sequence alignment and subsequent data analysis. Although Schoch, Seifert, Huhndorf, et al. (2012) concluded that ribosomal markers (e.g., ITS) have fewer problems with PCR amplification than protein‐coding markers (e.g., COI), the difficulties in generating a reliable alignment are an important drawback to the use of ITS as a DNA barcode marker (Dentinger et al., 2011; Seifert et al., 2007). Furthermore, sequence variation among paralogues can result in uncertain base calls. Despite these caveats, the availability of ITS sequences from a large number of fungal species in GenBank is a major advantage that often outweighs the complications introduced by alignment problems.

The current study suggests that the COI can be an additional barcode marker for particular taxonomic groups of fungi when ITS is unsuitable (e.g., some genera in Ascomycota or some species of mushrooms discussed in this study) or for examining fresh material through a cDNA based approach. However, this approach needs to be extended to determine its suitability for other fungi. Moreover, COI sequences generated phylogenetic groupings for *Pleurotus* similar to those for ITS while having the advantage of being easily aligned. These results justify the broader examination of cDNA‐based analysis to test the potential of COI as a barcode marker that could complement ITS, in much the same fashion that two gene regions (rbcL, matK) have been adopted as the standard barcode regions for plants (Hollingsworth et al., 2009). Future efforts should explore the use of COI in groups where ITS is unable to deliver species‐level resolution.

## CONFLICT OF INTEREST

None declared.

## AUTHOR CONTRIBUTIONS

Farhat A. Avin: designed research, performed research, analyzed data, wrote the paper; Subha Bhassu: project adviser, edited the paper, analysis adviser; Dr. Tan Yee Shin: project adviser, edited the paper, analysis adviser; Thomas W. A. Braukmann: analyzed data, edited the paper; Vikineswary Sabaratnam: project leader, project financial leader, project adviser, edited the paper; Paul Hebert: project adviser, edited the paper, analysis adviser.

## References

[ece33049-bib-0001] Avin, F. A. , Bhassu, S. , Rameeh, V. , Tan, Y. S. , & Vikineswary, S. (2016). Genetics and hybrid breeding of *Pleurotus pulmonarius*: Heterosis, heritability and combining ability. Euphytica, 209, 85–102.

[ece33049-bib-0002] Avin, F. A. , Bhassu, S. , & Sabaratnam, V. (2013). A simple and low‐cost technique of DNA extraction from edible mushrooms examined by molecular phylogenetics. Research on Crops, 14, 897–901.

[ece33049-bib-0003] Avin, F. A. , Bhassu, S. , Shin, T. Y. , & Sabaratnam, V. (2012). Molecular classification and phylogenetic relationships of selected edible Basidiomycetes species. Molecular Biology Reports, 39, 7355–7364. 22327649 10.1007/s11033-012-1567-2

[ece33049-bib-0004] Avin, F. A. , Bhassu, S. , Shin, T. Y. , & Vikineswary, S. (2014). DNA pedigree tracking to identify compatible mating partners of *Pleurotus pulmonarius* . Journal of Animal and Plant Sciences, 24, 89–97.

[ece33049-bib-0005] Avin, F. A. , Bhassu, S. , Tan, Y. S. , Shahbazi, P. , & Vikineswary, S. (2014). Molecular divergence and species delimitation of the cultivated oyster mushrooms: Integration of IGS1 and ITS. The Scientific World Journal, 2014, 1–11. 10.1155/2014/793414 PMC3918722 24587752

[ece33049-bib-0006] Bao, D. , Ishihara, H. , Mori, N. , & Kitamoto, Y. (2004). Phylogenetic analysis of oyster mushrooms (*Pleurotus* spp.) based on restriction fragment length polymorphisms of the 5′ portion of 26S rDNA. Journal of Wood Science, 50, 169–176.

[ece33049-bib-0007] Begerow, D. , Nilsson, H. , Unterseher, M. , & Maier, W. (2010). Current state and perspectives of fungal DNA barcoding and rapid identification procedures. Applied Microbiology and Biotechnology, 87, 99–108. 20405123 10.1007/s00253-010-2585-4

[ece33049-bib-0008] Blackwell, M. (2011). The Fungi: 1, 2, 3… 5.1 million species? American Journal of Botany, 98, 426–438. 21613136 10.3732/ajb.1000298

[ece33049-bib-0009] Dentinger, B. T. M. , Didukh, M. Y. , & Moncalvo, J. M. (2011). Comparing COI and ITS as DNA barcode markers for mushrooms and allies (*Agaricomycotina*). PLoS One, 6, e25081. 21966418 10.1371/journal.pone.0025081 PMC3178597

[ece33049-bib-0010] Excoffier, L. , Laval, G. , & Schneider, S. (2005). Arlequin (version 3.0): An integrated software package for population genetics data analysis. Evolutionary Bioinformatics Online, 1, 47–50. PMC2658868 19325852

[ece33049-bib-0011] Felsenstein, J. (1985). Confidence limits on phylogenies: An approach using the bootstrap. Evolution, 783–791. 28561359 10.1111/j.1558-5646.1985.tb00420.x

[ece33049-bib-0012] Férandon, C. , Moukha, S. , Callac, P. , Benedetto, J. P. , Castroviejo, M. , & Barroso, G. (2010). The *Agaricus bisporus cox1* gene: The longest mitochondrial gene and the largest reservoir of mitochondrial group I introns. PLoS One, 5, e14048. 21124976 10.1371/journal.pone.0014048 PMC2987802

[ece33049-bib-0013] Geiser, D. M. , Klich, M. A. , Frisvad, J. C. , Peterson, S. W. , Varga, J. , & Samson, R. A. (2007). The current status of species recognition and identification in Aspergillus. Studies in Mycology, 59, 1–10. 18490947 10.3114/sim.2007.59.01 PMC2275194

[ece33049-bib-0014] Gilmore, S. R. , Graefenhan, T. , Louis Seize, G. , & Seifert, K. A. (2009). Multiple copies of cytochrome oxidase 1 in species of the fungal genus *Fusarium* . Molecular Ecology Resources, 9, 90–98. 21564969 10.1111/j.1755-0998.2009.02636.x

[ece33049-bib-0015] Gonzalez, P. , Barroso, G. , & Labarère, J. (1998). Molecular analysis of the split cox1 gene from the Basidiomycota *Agrocybe aegerita*: Relationship of its introns with homologous Ascomycota introns and divergence levels from common ancestral copies. Gene, 220, 45–53. 9767103 10.1016/s0378-1119(98)00421-1

[ece33049-bib-0016] Hall, T. A. (1999). BioEdit: a user‐friendly biological sequence alignment editor and analysis program for Windows 95/98/NT Nucleic Acids Symposium Series, 41, 95–98.

[ece33049-bib-0017] Haridas, S. , & Gantt, J. S. (2010). The mitochondrial genome of the wood‐degrading basidiomycete *Trametes cingulata* . FEMS Microbiology Letters, 308, 29–34. 20455947 10.1111/j.1574-6968.2010.01979.x

[ece33049-bib-0018] Hebert, P. D. N. , Cywinska, A. , & Ball, S. L. (2003). Biological identifications through DNA barcodes. Proceedings of the Royal Society of London. Series B: Biological Sciences, 270, 313–321. 12614582 10.1098/rspb.2002.2218 PMC1691236

[ece33049-bib-0019] Hollingsworth, P. M. , Forrest, L. L. , Spouge, J. L. , Hajibabaei, M. , Ratnasingham, S. , Van Der Bank, M. , … Fazekas, A. J. (2009). A DNA barcode for land plants. Proceedings of the National Academy of Sciences, 106, 12794–12797. 10.1073/pnas.0905845106 PMC2722355 19666622

[ece33049-bib-0020] James, T. Y. , Kauff, F. , Schoch, C. L. , Matheny, P. B. , Hofstetter, V. , Cox, C. J. , … Miadlikowska, J. (2006). Reconstructing the early evolution of Fungi using a six‐gene phylogeny. Nature, 443, 818–822. 17051209 10.1038/nature05110

[ece33049-bib-0021] Lahaye, R. , Van Der Bank, M. , Bogarin, D. , Warner, J. , Pupulin, F. , Gigot, G. , … Savolainen, V. (2008). DNA barcoding the floras of biodiversity hotspots. Proceedings of the National Academy of Sciences, 105, 2923–2928. 10.1073/pnas.0709936105 PMC2268561 18258745

[ece33049-bib-0022] Lanave, C. , Preparata, G. , Sacone, C. , & Serio, G. (1984). A new method for calculating evolutionary substitution rates. Journal of Molecular Evolution, 20, 86–93. 6429346 10.1007/BF02101990

[ece33049-bib-0023] Librado, P. , & Rozas, J. (2009). DnaSP v5: A software for comprehensive analysis of DNA polymorphism data. Bioinformatics, 25, 1451–1452. 19346325 10.1093/bioinformatics/btp187

[ece33049-bib-0024] Nguyen, H. D. T. , & Seifert, K. A. (2008). Description and DNA barcoding of three new species of *Leohumicola* from South Africa and the United States. Persoonia: Molecular Phylogeny and Evolution of Fungi, 21, 57–69. 10.3767/003158508X361334 PMC2846127 20396577

[ece33049-bib-0025] Pawlik, A. , Janusz, G. , Koszerny, J. , Małek, W. , & Rogalski, J. (2012). Genetic diversity of the edible mushroom *Pleurotus* sp. by amplified fragment length polymorphism. Current Microbiology, 65, 438–445. 22767319 10.1007/s00284-012-0175-7 PMC3426667

[ece33049-bib-0026] Ratnasingham, S. , & Hebert, P. D. N. (2007). BOLD: The barcode of life data system (http://www.barcodinglife.org). Molecular Ecology Notes, 7, 355–364. 18784790 10.1111/j.1471-8286.2007.01678.x PMC1890991

[ece33049-bib-0027] Robert, V. , Szöke, S. , Eberhardt, U. , Cardinali, G. , Meyer, W. , Seifert, K. A. , … Lewis, C. T. (2011). The quest for a general and reliable fungal DNA barcode. Open Applied Informatics Journal, 5, 45–61.

[ece33049-bib-0028] Rozen, S. , & Skaletsky, H. (2000). Primer3 on the WWW for general users and for biologist programmers. Methods Molecular Biology, 132, 365–386. 10.1385/1-59259-192-2:365 10547847

[ece33049-bib-0029] Schoch, C. L. , & Seifert, K. A. (2012). Reply to Kiss: Internal transcribed spacer (ITS) remains the best candidate as a universal DNA barcode marker for Fungi despite imperfections. Proceedings of the National Academy of Sciences, 109, E1812. 10.1073/pnas.1117018109 PMC3341068 22454494

[ece33049-bib-0030] Schoch, C. L. , Seifert, K. A. , Caldeira, K. , Myhrvold, N. P. , Alvarez, R. A. , Pacala, S. W. , … Mullins, R. D. (2012). Limits of nuclear ribosomal DNA internal transcribed spacer (ITS) sequences as species barcodes for Fungi. Proceedings of the National Academy of Sciences, 109, 10741–10742. 10.1073/pnas.1207143109 PMC3390822 22715287

[ece33049-bib-0031] Schoch, C. L. , Seifert, K. A. , Huhndorf, S. , Robert, V. , Spouge, J. L. , Levesque, C. A. , … Crous, P. W. (2012). Nuclear ribosomal internal transcribed spacer (ITS) region as a universal DNA barcode marker for Fungi. Proceedings of the National Academy of Sciences, 109, 6241–6246. 10.1073/pnas.1117018109 PMC3341068 22454494

[ece33049-bib-0032] Schwarz, G. (1978). Estimating the dimension of a model. The Annals of Statistics, 6, 461–464.

[ece33049-bib-0033] Seifert, K. A. (2009). Progress towards DNA barcoding of fungi. Molecular Ecology Resources, 9, 83–89. 10.1111/j.1755-0998.2009.02635.x 21564968

[ece33049-bib-0034] Seifert, K. A. , Samson, R. A. , DeWaard, J. R. , Houbraken, J. , Levesque, C. A. , Moncalvo, J. M. , … Hebert, P. D. N. (2007). Prospects for fungus identification using CO1 DNA barcodes, with *Penicillium* as a test case. Proceedings of the National Academy of Sciences, 104, 3901–3906. 10.1073/pnas.0611691104 PMC1805696 17360450

[ece33049-bib-0035] Stielow, J. , Lévesque, C. , Seifert, K. , Meyer, W. , Iriny, L. , Smits, D. , … Chaduli, D. (2015). One fungus, which genes? Development and assessment of universal primers for potential secondary fungal DNA barcodes. Persoonia: Molecular Phylogeny and Evolution of Fungi, 35, 242–263. 10.3767/003158515X689135 PMC4713107 26823635

[ece33049-bib-0036] Tamura, K. (1992). Estimation of the number of nucleotide substitutions when there are strong transition‐transversion and G+ C‐content biases. Molecular Biology and Evolution, 9, 678–687. 1630306 10.1093/oxfordjournals.molbev.a040752

[ece33049-bib-0037] Tamura, K. , Stecher, G. , Peterson, D. , Filipski, A. , & Kumar, S. (2013). MEGA6: Molecular evolutionary genetics analysis version 6.0. Molecular Biology and Evolution, 30, 2725–2729. 24132122 10.1093/molbev/mst197 PMC3840312

[ece33049-bib-0038] Vialle, A. , Feau, N. , Allaire, M. , Didukh, M. , Martin, F. , Moncalvo, J. , & Hamelin, R. C. (2009). Evaluation of mitochondrial genes as DNA barcode for Basidiomycota. Molecular Ecology Resources, 9, 99–113. 21564970 10.1111/j.1755-0998.2009.02637.x

[ece33049-bib-0039] Vilgalys, R. , Moncalvo, J. M. , Liou, S. R. , & Volovsek, M. (1996). Recent advances in molecular systematics of the genus *Pleurotus* In Royse D. J. (Ed.), Mushroom biology and mushroom products: Proceedings of the 2nd international conference (pp. 91–101). Pennsylvania State University: World Society for Mushroom Biology and Mushroom Products: PA (USA).

[ece33049-bib-0040] Wang, Y. , Zeng, F. , Hon, C. , Zhang, Y. , & Leung, F. (2008). The mitochondrial genome of the Basidiomycete fungus *Pleurotus ostreatus* (oyster mushroom). FEMS Microbiology Letters, 280, 34–41. 18248422 10.1111/j.1574-6968.2007.01048.x

[ece33049-bib-0041] White, T. J. , Bruns, T. , Lee, S. , & Taylor, J. (1990). PCR protocols: A guide to methods and applications. New York, NY: Academic Press.

[ece33049-bib-0042] Ye, J. , Coulouris, G. , Zaretskaya, I. , Cutcutache, I. , Rozen, S. , & Madden, T. (2012). Primer‐BLAST: A tool to design target‐specific primers for polymerase chain reaction. BMC Bioinformatics, 13, 134–144. 22708584 10.1186/1471-2105-13-134 PMC3412702

[ece33049-bib-0043] Zervakis, G. , & Balis, C. (1996). A pluralistic approach in the study of *Pleurotus* species with emphasis on compatibility and physiology of the European morphotaxa. Mycological Research, 100, 717–731.

